# Anterior Jugular Vein Hemangioma: A Diagnostic Conundrum and Report of a Rare Case

**DOI:** 10.1055/s-0042-1759514

**Published:** 2022-12-02

**Authors:** Vivek Dokania, Shashikant Mhashal, Devkumar Rengaraja, Prashant Kewle

**Affiliations:** 1 Department of Otolaryngology- Head & Neck Surgery, HBT Medical College and Dr RN Cooper Municipal General Hospital, Juhu, Mumbai, Maharashtra, India; 2 Department of Otolaryngology- Head & Neck Surgery, Fortis Hiranandani Hospital, Navi Mumbai, Mumbai, Maharashtra, India; 3 Department of Otolaryngology- Head & Neck Surgery, Sushrut Hospital and Research Center, Chembur, Mumbai, Maharashtra, India

**Keywords:** anterior jugular vein, hemangioma, neck mass, case report

## Abstract

Hemangioma is a common tumor accounting for 8 to 10 % of benign neoplasm. However, hemangioma arising from blood vessels is rare and even rarer if the vessel involved is anterior jugular vein (AJV). AJV hemangioma can be confused with jugular phlebectasia, laryngocele, thyroglossal cyst, simple cyst, or other vascular malformation of same origin. They should be considered in differential of midline/paramedian neck swelling. Surgical resection is the treatment of choice whenever possible, and even allows for histopathological evaluation and a confirmatory diagnosis. Being an extremely rare entity, there is paucity in literature about it and more publications are required to extend understanding and eliminate existing doubts about the pathology. We present an extremely rare case of AJV hemangioma that presented as a painless midline swelling and was initially confused as simple neck cyst on radiological assessment. We believe that this is the second only case of AJV hemangioma reported in English literature.

Vascular tumors and anomalies can present as solitary neck swelling in adults. Their designation into hemangiomas or vascular malformations has never been easy, since more classification systems have been proposed over the years, as these anomalies have been thoroughly studied. 1 2 As a primary tumor, hemangioma that originates from vessel wall and particularly in the wall of anterior jugular vein (AJV) is extremely rare, while that arising from the wall of external jugular vein (EJV) is only slightly more common. To the best of our knowledge, there are only six reports of vascular hemangioma arising from EJV, 3 4 5 6 7 8 and only one prior report of AJV hemangioma in English literature. 9 We present a case of AJV hemangioma and believe that this is the second only case of AJV hemangioma reported in the English literature.

## Case Report

A 36-year-old female presented with a complaint of painless midline neck swelling. She first noted a small nodular swelling 6 months before, which had gradually increased in size. She was otherwise healthy and did not report a history of alcohol consumption or of smoking. No genetic or syndromic abnormalities were reported from her family. There was no history of preceding neck trauma, surgery, or upper respiratory tract infection.

On physical examination, there was a nonpulsatile, soft, and nontender left paramedian neck swelling that moved slightly with deglutition. Overlying skin illustrated no erythema, warmth, or tenderness. No cervical lymphadenopathy was noted. Her laboratory reports including complete blood count, basic metabolic panel, chest X-ray, and electrocardiography were unremarkable. On ultrasonography, a well-defined multiseptate, cystic lesion measuring 5.5 × 2.4 cm seen within the subcutaneous tissue in the left cervical region, anterior to the thyroid gland. A simple neck cyst was suspected on radiological assessment. Being uncertain about the diagnosis, the lesion was explored under general anesthesia.

**Fig. 1:**
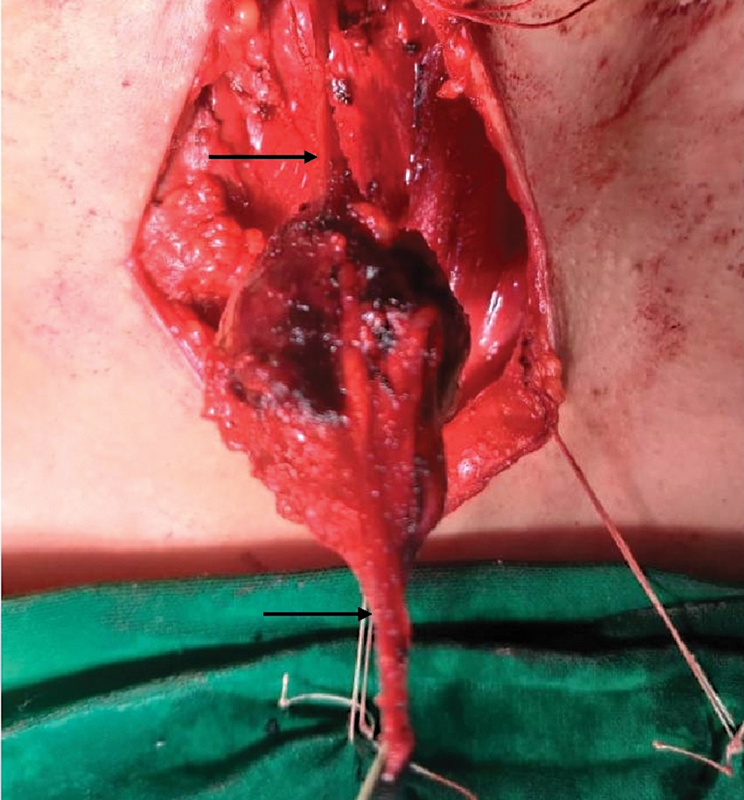
Tumor arising from the anterior jugular vein (arrow).

A surgical exploration was undertaken under general anesthesia with oral intubation using flexometallic endotracheal tube and following anesthesia medications: premedication (injection midazolam 1 gm, injection Emeset 4 mg, and injection glycopyrrolate 0.2 mg), induction (injection propofol 100 mg and injection succinylcholine 100 mg), relaxant (injection cisatracurium 10 mg), and analgesia (injection fentanyl 100 µg and injection paracetamol 1 gm). Patient was kept in supine position with neck slightly extended and a horizontal skin crease incision was taken. Subplatysmal skin flaps were raised both superiorly and inferiorly. A mass arising from the left AJV was isolated after careful dissection ( Fig. 1 ). The mass was excised after ligating the AJV. The tumor appeared globular, reddish, and did not seem to infiltrate the surrounding structure. A surgical drain was kept and suturing was done in two layers. The postoperative period was uneventful. Drain was removed on 3rd postoperative day and sutures were removed on 10th day, displaying a healthy wound.

**Fig. 2:**
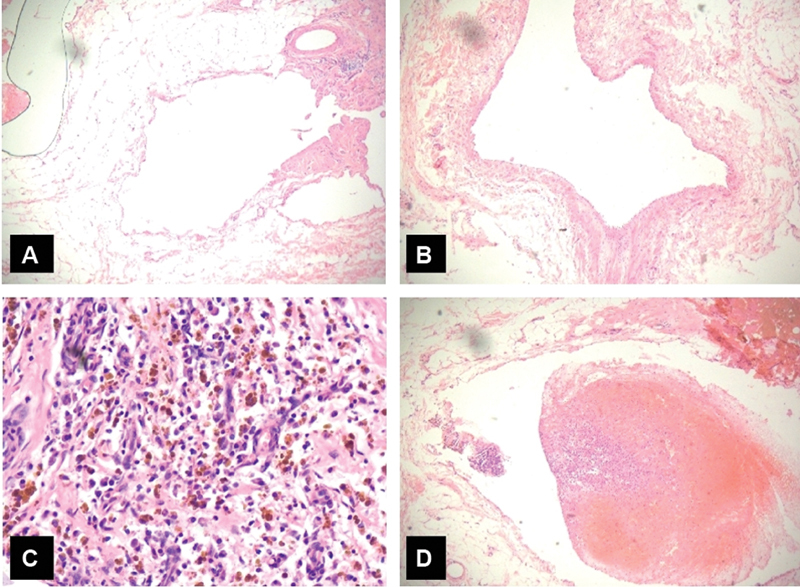
Histopathological examination findings. ( **A** ) Thin-walled blood vessels with focal wall thickening. ( **B)** Attenuated muscle layer in large-caliber blood vessel. ( **C** ) Siderophages and histiocytes in wall. **(D** ) Thrombus in large-caliber blood vessel.

**Fig. 3:**
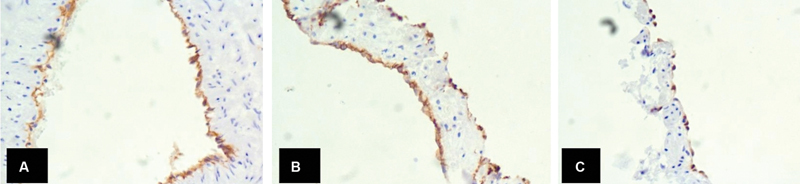
Immunohistochemical examination findings. ( **A** ) Endothelial cells CD31 positive. ( **B** ) Endothelial cells CD34 positive. ( **C** ) Endothelial cells ERG positive.

The histopathological examination of resected tissue showed collection of abnormal veins of varying sizes and proportions. Some vessels are large and thin walled whereas others showed compressed smooth vessel in the wall. Foci of wall thickening with infiltration by siderophages, histiocytes, and lymphocytes were noted. Organized thrombi were also noted ( Fig. 2A–D ). The endothelial lining of the large vessels was immunopositive for CD31, CD34, and ERG ( Fig. 3A–C ). Based on these features, a confirmatory diagnosis of AJV hemangioma was made. No recurrence or complication has been noted until 1-year postoperative follow-up.

## Discussion

A hemangioma is a usually benign vascular tumor derived from blood vessel cell types. There are various types of hemangiomas. The more common types include capillary, cavernous, combined, and lobular capillary types. There are very few reports of primary venous hemangiomas in the literature. Some authors consider these lesions to be present since birth, while others consider it to be an acquired process caused by trauma, sepsis, pressure changes, and hormonal alterations. 10

In contrary to various childhood vascular tumors, venous hemangiomas, like the one in present report, do not show signs of involution. 10 In terms of anatomical location, head and neck hemangiomas account for 60% of cases, while 25% involve the trunk and 15% the extremities. 1

As a primary tumor, hemangioma that originates from the vessel wall and particularly in the wall of EJV or AJV is extremely rare, while that arising from the wall of AJV is slightly more uncommon. The first report of preoperative diagnosed case of hemangioma arising from EJV was reported by Meyers in 1967. 3 Subsequently, to be the best of our knowledge, five more reports of hemangioma arising from EJV were reported in English literature. 4 5 6 7 8

We report a particularly rare case of hemangioma arising from AJV. We believe that this is the second case of AJV hemangioma reported in English literature; the only prior case of AJV hemangioma was reported by Irfan et al in 2010. 9 Similar to our case, there was a radiological diagnostic dilemma with simple neck cyst and a final diagnosis was achieved after surgical excision.

Treatment options include watchful waiting, sclerotherapy, embolization, and surgical excision. 8 Surgical excision with ligation of the vessel is the treatment of choice as it allows for a confirmatory histopathological diagnosis and has zero reports of recurrence/relapse; however, it is generally advocated for tumor causing compressive symptoms or cosmetic deformity.

Differentials of AJV hemangioma/EJV hemangioma include jugular phlebectasia, laryngocele, thyroglossal cyst, branchial cyst, simple cyst, or other vascular malformation of same origin, including aneurysms. 11

## Conclusion

AJV hemangioma is an extremely rare lesion and should be considered in differential diagnosis of midline/paramedian neck swelling. They can be confused with jugular phlebectasia, laryngocele, thyroglossal cyst, simple cyst, or other vascular malformation of same origin. Clinical examination and radiological assessment can aid in diagnosis and surgical planning; however, they can sometimes cause diagnostic dilemma with close differentials. Surgical excision is the preferred treatment whenever possible, and even allows for histopathological evaluation and a confirmatory diagnosis. Because of its rarity, a comprehensive data is lacking and more publications are required to have a better understanding about the pathophysiology, presentation, and management of this tumor.

## References

[1] Mulliken J B Glowacki J Hemangiomas and vascular malformations in infants and children: a classification based on endothelial characteristics Plast Reconstr Surg 1982 69 03 412 422 7063565 10.1097/00006534-198203000-00002

[2] Steiner J E Drolet B A Classification of vascular anomalies: an update Semin Intervent Radiol 2017 34 03 225 232 28955111 10.1055/s-0037-1604295 PMC5615389

[3] Meyers M A Hemangioma of the external jugular vein Radiology 1967 89 03 483 485 6034916 10.1148/89.3.483

[4] Sarteschi L M Bonanomi G Mosca F Ferrari M External jugular vein hemangioma occurring as a lateral neck mass J Ultrasound Med 1999 18 10 719 721 10511307 10.7863/jum.1999.18.10.719

[5] Ahuja A T Yuen H Y Wong K T External jugular vein vascular malformation: sonographic and MR imaging appearances AJNR Am J Neuroradiol 2004 25 02 338 342 14970043 PMC7974618

[6] Lin Y C Tsai Y T Lin C Y Lee C Y Hong G J Tsai C S Haemangioma arising from external jugular vein ANZ J Surg 2010 80 10 755 756 21040340 10.1111/j.1445-2197.2010.05465.x

[7] Yorgancılar A E Kınış V Gün R Bakır S Ozbay M Meriç F Hemangioma arising from external jugular vein mimicking neck mass Kulak Burun Bogaz Ihtis Derg 2012 22 04 241 244 22770262 10.5606/kbbihtisas.2012.047

[8] de Oliveira J CP Barreto F TR Chimelli B CAR External jugular vein hemangioma: case report J Vasc Bras 2019 18 e20180026 10.1590/1677-5449.180026 31320886 PMC6629457

[9] Irfan M Idayu M Y Venkatesh R N Cavernous hemangioma mimicking anterior jugular vein phlebectasia Med J Malaysia 2010 65 01 68 69 21265254

[10] Werner J A Dünne A A Folz B J Current concepts in the classification, diagnosis and treatment of hemangiomas and vascular malformations of the head and neck Eur Arch Otorhinolaryngol 2001 258 03 141 149 11374256 10.1007/s004050100318

[11] Essa R A Ahmed S K Bapir D H Rasul S A Abubakr C P Hamad S Q Thrombosed external jugular vein aneurysm mimics to a branchial cyst: a novel case from Iraq and review of the literature Ann Med Surg (Lond) 2021 67 102533 34257963 10.1016/j.amsu.2021.102533 PMC8260851

